# Transcriptomic Profile of Genes Regulating Cellular Response to Extra- and Intracellular Stimuli in Porcine Ovarian Granulosa Cells During In Vitro Cultivation

**DOI:** 10.3390/ijms27125445

**Published:** 2026-06-16

**Authors:** Krzysztof Data, Wiesława Kranc, Małgorzata Blatkiewicz, Małgorzata Józkowiak, Magdalena Kulus, Jakub Kulus, Michał Gnus, Dominika Domagała, Piotr Paweł Chmielewski, Anna Kałuża, Agnieszka Żok, Julia Niebora, Artur Bryja, Anna Olechnowicz, Hanna Piotrowska-Kempisty, Paul Mozdziak, Bartosz Kempisty, Paweł Antosik, Dorota Bukowska, Mariusz T. Skowroński

**Affiliations:** 1 Division of Anatomy, Department of Human Morphology and Embryology, Faculty of Medicine, Wroclaw Medical University, 50-368 Wroclaw, Poland; krzysztof.data@umw.edu.pl (K.D.); dominika.domagala@umw.edu.pl (D.D.); piotr.chmielewski@umw.edu.pl (P.P.C.); julia.niebora@umw.edu.pl (J.N.); artur.bryja@umw.edu.pl (A.B.); bartosz.kempisty@umw.edu.pl (B.K.); 2 Department of Anatomy, Poznan University of Medical Sciences, 60-781 Poznan, Poland; wkranc@ump.edu.pl; 3 Department of Histology and Embryology, Poznan University of Medical Sciences, 60-812 Poznan, Poland; mblatkiewicz@ump.edu.pl (M.B.); aolechnowicz@ump.edu.pl (A.O.); 4 Department of Toxicology, Poznan University of Medical Sciences, 60-812 Poznan, Poland; malgorzata.jozkowiak@umw.edu.pl (M.J.); hpiotrow@ump.edu.pl (H.P.-K.); 5 Department of Veterinary Surgery, Institute of Veterinary Medicine, Nicolaus Copernicus University in Torun, 87-100 Torun, Poland; magdalena.kulus@umk.pl (M.K.); michalgnus@umk.pl (M.G.); pantosik@umk.pl (P.A.); 6 Department of Diagnostics and Clinical Sciences, Institute of Veterinary Medicine, Nicolaus Copernicus University in Torun, 87-100 Torun, Poland; jakub.kulus@umk.pl (J.K.); dbukowska@umk.pl (D.B.); 7 Department of Biochemistry and Immunochemistry, Division of Chemistry and Immunochemistry, Wroclaw Medical University, 50-369 Wroclaw, Poland; anna.kaluza@umw.edu.pl; 8 Division of Philosophy of Medicine and Bioethics, Poznan University of Medical Sciences, 60-812 Poznan, Poland; agzok@ump.edu.pl; 9 Department of Basic and Preclinical Science, Institute of Veterinary Medicine, Nicolaus Copernicus University in Torun, 87-100 Torun, Poland; 10 Prestage Department of Poultry Science, North Carolina State University, Raleigh, NC 27695, USA; pemozdzi@ncsu.edu; 11 Physiology Graduate Faculty, North Carolina State University, Raleigh, NC 27695, USA; 12 Department of Obstetrics and Gynecology, University Hospital and Masaryk University, 602 00 Brno, Czech Republic

**Keywords:** ovarian follicle, folliculogenesis, steroidogenesis, signaling pathway, intercellular communication

## Abstract

Granulosa cells (GCs), an element of the ovarian follicle, are crucial for oocyte maturation, folliculogenesis, and steroidogenesis. Granulosa cells play a crucial role in fertilization by providing metabolic and hormonal support to the oocyte, maintaining its quality and regulating its meiotic arrest. Oocyte quality and fertilization efficiency depend on the proper activity of GCs, especially their mutual communication, providing metabolic support and protecting against oxidative stress. When interrupted, they may take part in the pathogenesis of polycystic ovary syndrome, premature ovarian failure, primary ovarian insufficiency, and diminished ovarian reserve. GCs are enclosed in the antrum where they communicate with surrounding cells, create a dynamic microenvironment, and regulate hormone biosynthesis. To analyze molecular mechanisms regulating endogenous signaling, it is important to consider the dynamic transcriptomic response of porcine GCs during in vitro culturing over 48, 96, and 144 h. Transcriptomic analysis revealed a variable and dynamic transcriptional upregulation of genes associated with cellular response to endogenous and external stimuli, chemical compound metabolism, vascular development, and GCs migration. Also, proven by Gene Ontology (GO) enrichment analysis, the following terms were highlighted: “cellular response to chemical stimulus” and “cellular response to organic substance”. Specific genes, such as *HSD3B1*, *POSTN*, *LOX*, *SERPINB2*, *ITGB3*, *ANKRD1*, *SLC1A1*, and *SFRP2*, exhibited significant expression changes, suggesting extensive GCs self-regulation and metabolism changes. Further analysis indicates improvements in cellular response to a cytokine stimulus, growth factor response, hormone response, enzyme-linked receptor protein signaling, and positive regulation of cell migration. These findings suggest interweaving of regulatory mechanisms underlying intercellular communication in GCs during in vitro culturing, despite the lack of signals from the native ovarian environment. Further investigating interplays of detecting pathways will provide a more comprehensive understanding and even insights into the potential clinical use of the knowledge about the role of GCs in folliculogenesis, oocyte maturation and ovulation.

## 1. Introduction

Granulosa cells (GCs) are somatic cells surrounding the oocyte, taking a part at forming the ovarian follicle [1]. In the advanced stage of folliculogenesis, while the ovarian follicle is in the pre-ovulatory phase, GCs surround the antrum, which is filled with follicular fluid (FF). Additionally, GCs are in direct connection with cumulus cells (CCs), which envelop oocytes [1,2,3]. CCs are a secreting, cumulus expansion-enabling factor and hyaluronic acid during the ovulatory phase, resulting in follicle rupture and finally ovulation [4,5]. Interaction between oocytes and CCs is possible even though the zona pellucida, a thin layer of gelatinous extracellular matrix, separates the cells [6,7]. The last subpopulation of follicular cells are theca cells, forming the most external cellular layer of the follicle, providing structural support and supplying nutrients to GCs and oocytes [8,9]. The layer of GCs constitutes a highly dynamic microenvironment essential for regulating oocyte maturation, folliculogenesis, and steroidogenesis, all of which are fundamental for reproductive success [10,11,12]. The metabolism of the single cell and the structure of the GCs layer rearrange during folliculogenesis; thus, continuous proper development and response to endogenous and external stimuli are essential for the ovarian cycle [13,14,15]. GCs play a central role in hormone biosynthesis, especially the aromatization and secretion of estrogens and progesterone, which are afterwards secreted into the follicular fluid [3,16,17]. These hormones influence the developing follicle, affecting both local follicular dynamics and systemic reproductive function, also regulating proper functioning and secreting the profile of other reproductive organs [15,18,19]. Estrogen regulates activation of its own receptor, which is a key transcription factor in the maintenance of GCs growth, differentiation, and the ovulation function of ovarian follicles and oocytes [20,21]. Furthermore, the regulated proliferation and differentiation of GCs influence follicular growth and maturation, processes crucial for oocyte development and ovulation [22]. An integral component of the response to endogenous stimuli is the involvement of catalytic processes. Catalytic processes, driven by a wide spectrum of various enzymes, are fundamental for every aspect of the follicular environment. For instance, cytochrome P450 enzymes, such as CYP19A1 (aromatase), are crucial for steroidogenesis, converting androgens to estrogens at GCs and influencing hormonal regulation of the reproductive cycle [23]. Similarly, 17α-hydroxylase and 3β-hydroxysteroid dehydrogenase are essential for the production of precursors in the steroidogenic pathway [24,25]. Beyond steroidogenesis, catalytic enzymes regulate follicular ECM remodeling, a dynamic process necessary for follicle development, oocyte maturation and ovulation [26,27]. Enzymes such as matrix metalloproteinases (MMPs) degrade ECM components to facilitate follicular rupture and release of the mature oocyte [28,29]. Proteolytic enzymes such as plasminogen activators contribute to this remodeling by converting plasminogen to plasmin, the protease that further aids ECM breakdown [30,31].

The physiological status of cells, also GCs, can be rated via transcriptomic analysis, which offers insights into the molecular landscape of GCs, enabling the characterization of gene expression profiles across the time points of functional and developmental states [32,33,34,35]. The methodology allows for identifying and characterizing crucial regulatory pathways associated with cellular proliferation, differentiation, and metabolic adaptation, affording insights into the dynamically modulated activity of GCs during folliculogenesis [36,37,38]. The detected signaling pathways and enriched genes may be assigned to complex processes in which they perform a regulatory role, stimulating metabolism and activity of cells [39,40]. Endogenous stimulation through growth factors and signaling pathways is critical for GCs metabolism, also modulating to the metabolic and enzymatic pathways determining energy acquisition, nutrient processing, and secreting of whole ovarian follicles [41,42,43,44,45]. Understanding the interplay between growth factors and Wnt pathways resulting in influencing steroidogenesis, cytokine production, ECM dynamics, and apoptosis is crucial for discovering the molecular basics of reproductive processes [46,47,48,49,50].

One of the tools to reach molecular basics is in vitro culturing systems, providing a controlled environment to study GCs behavior and enabling precise manipulation of experimental environments [51,52,53,54]. Such models, particularly using porcine GCs due to their physiological similarity to human models, offer valuable insights into independent GCs gene regulatory networks, facilitating translational research into fertility treatments and therapeutic strategies [55,56,57]. In vitro studies are a fundamental base to understand the broad interdependencies of cell biology. Thus, the molecular pattern of a single cell may have an impact on the functioning of the entire cellular complex. As GCs are main cells that respond to FSH and LH, controlling follicular development and ovulation by producing progesterone and estrogen places them at the center of ovarian biology [58]. Isolating GCs from extensive intercellular connections will allow for the study of biological mechanisms under controlled conditions.

The objective of the study was to undertake a comprehensive transcriptomic analysis of porcine GCs cultured in vitro at various points in time to discover the molecular mechanisms regulating endogenous signaling. The rationale for the study was to identify novel molecular markers as potential therapeutic targets for ovarian disorders, advancing clinical applications in assisted reproductive technologies. Proper understanding of GCs’ response to in vitro culture conditions will enable us to project and propose proper conditions for in vitro research models, including platforms for drug testing, targeted therapies, and diagnostics.

## 2. Results

**Figure 1:**
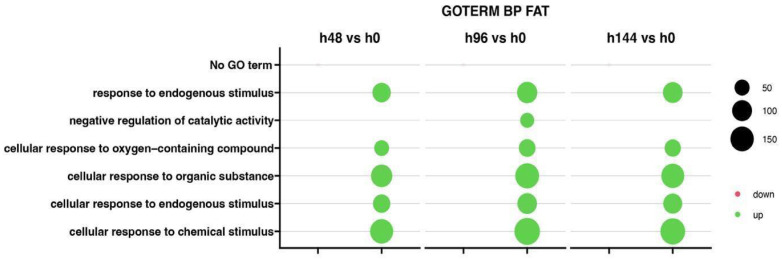
A bubble plot of the overrepresented gene sets in the DAVID GO PB DIRECT annotation database, derived from comparisons of gene expression profiles between 48 h, 96 h, and 144 h versus the control (0 h). The graph shows selected ontology groups related to the regulation of endogenous stimulation and metabolism of chemical compounds. Only GO groups surpassing established criteria (*p*-value with correction < 0.05 and a minimal number of genes per group > 2) are depicted as colored bubbles. The size of each bubble corresponds to the number of differentially expressed genes associated with the GO biological process terms. Bubble transparency indicates the *p*-value, with greater transparency suggesting proximity to the *p* = 0.05 cut-off value. Green bubbles represent overexpressed genes.

The “Limma” algorithm identified differentially expressed genes (DEGs) between experimental groups. Gene ontology (GO) biological process analysis was performed (Figure 1). Five of the six highlighted processes were significantly upregulated at all of the time points of the experiment. The one GO group “negative regulation of catalytic activity” was enhanced only for the 96 h group. The highest number of enriched genes (150 genes) were indicated for “cellular response to chemical stimulus” and “cellular response to organic substance”.

**Figure 2:**
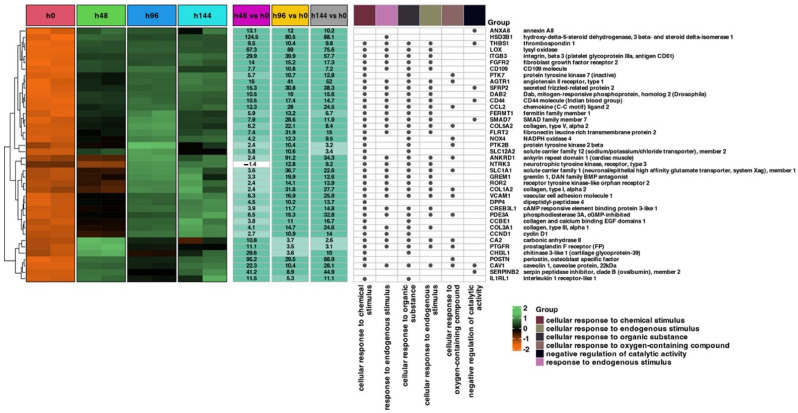
A heatmap showing the hierarchical clustering of genes with differential expression over 48, 96 and 144 h of the experiment compared to the control (0 h). Genes from the most significantly enriched GO groups, as determined by the lowest adjusted *p*-values, are represented by dark dots. Expression values are row-scaled and presented using a color spectrum ranging from red (indicating low expression) to green (indicating high expression). Only genes above a 10-fold change in expression are shown on the heatmap.

Gene expression patterns across conditions were visualized though a hierarchical clustering heatmap (Figure 2). The analysis revealed that the transcriptomic profile of genes involved in endogenous stimulation and chemical compound metabolism was activated during the experiment. The highest fold change was at the first time point of the experiment (48 h) for *HSD3B1*, *POSTN*, *LOX*, *SERPIN2B*, and *ITGB3* (fold change of 124.5, 95.2, 67.3, 41.2, and 29.9 respectively). The second time point (96 h) indicates *ANKRD1*, *HSD3B1*, *ITGB3*, *SLC1A1*, and *SFRP2* (fold change of 91.2, 80.5, 39.9, 36.7, and 30.8 respectively). The last of the analyzed time points (144 h) indicates that the highest fold-change expression changes were revealed for *POSTN*, *HSD3B1*, *LOX*, *ITGB3*, and *SERPINB2* (fold change of 88.9, 88.1, 75.6, 57.7, and 44.9 respectively). The lowest number of genes were involved in negative regulation of catalytic activity (7 genes).

**Figure 3:**
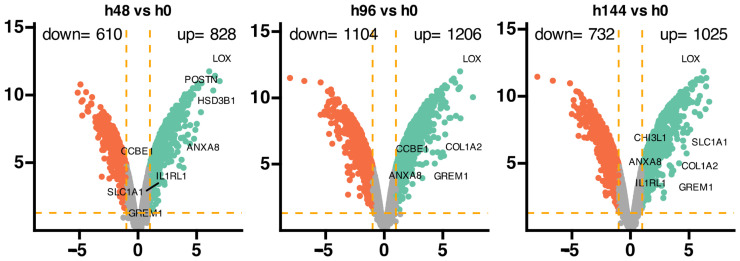
A volcano plot showing general expression profiles, where each point represents the mean expression of a single gene from a normalized microarray study. The orange dotted lines indicate the cut-off values determined by the following criteria: |fold change| = 2 and *p*-value = 0.05. Genes exceeding these cut-off lines are classified as differentially expressed and are shown as orange dots (down-regulated) and green dots (upregulated). The total number of up- and down-regulated genes is shown in the upper right and upper left corners. The compared groups are indicated above each graph (h48 vs. h0 (control); h96 vs. h0 (control); h144 vs. h0 (control)). In addition, symbols of the most differentially expressed endogenous stimulation and compound metabolism genes from each comparison are shown on the plots.

Volcano plots identify 610 down-regulated and 828 upregulated genes for the 48 h group (Figure 3). The most enriched genes related to endogenous stimulation and compound metabolism were *LOX*, *POSTN*, *HSD3B1*, *ANXA8*, *GREM1*, *CCBE1*, and *SLC1A1*. In the 96 h group, 1014 genes were inhibited, and 1206 genes were activated, with *LOX*, *COL1A2*, *GREM1*, *CCBE1*, and *ANXA8* being the most enriched genes related to endogenous stimulation and compound metabolism. The last time point (144 h) analysis indicates 732 inhibited and 1025 upregulated genes (genes related to endogenous stimulation and compound metabolism: *LOX*, *COL1A2*, *CHI3L1*, *SLC1A1*, *IL1RL1*, and *GREM1*). In summary, at all time points analyzed, the expression of *LOX*, *ANXA8*, and *GREM1* was increased.

**Figure 4:**
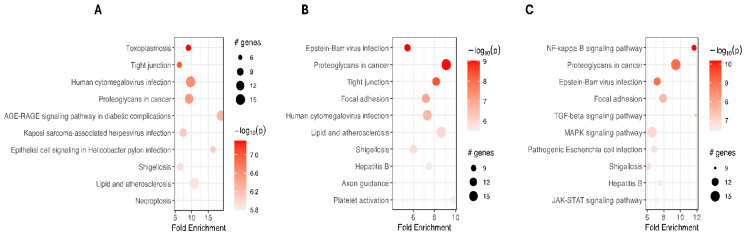
A bubble chart displaying enrichment results from experiments conducted at 48 h (**A**), 96 h (**B**), and 144 h (**C**) compared to the control (0 h). The horizontal axis represents fold enrichment values, while the vertical axis denotes enriched terms. Bubble size indicates the number of significant genes associated with each enriched term. Bubble color is determined by the −log_10_ value (lowest *p*-value), with shades closer to red indicating a higher level of significance in the enrichment. (abb. # genes, number of genes).

**Figure 5:**
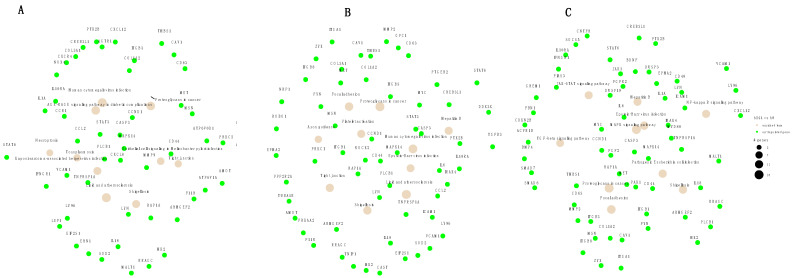
A graph representing the relationships between terms and genes, highlighting significant genes associated with enriched terms at 48 h (**A**), 96 h (**B**), and 144 h (**C**) compared to the control. Dot sizes are scaled based on the number of genes within each term, according to the legend. Green dots identify upregulated genes, while beige points enriched terms (abb. # genes, number of genes).

PathfindR was used to identify enriched pathways and active subnetworks within a protein–protein interaction network (Figure 4 and Figure 5). The analysis revealed that for all analyzed groups, “proteoglycan in cancers” was mainly enriched (Figure 4). Moreover, exploration of the associations between terms and significant genes have shown that all genes show increased expression, regardless of the group analyzed or the enrichment process (Figure 5).

**Figure 6:**
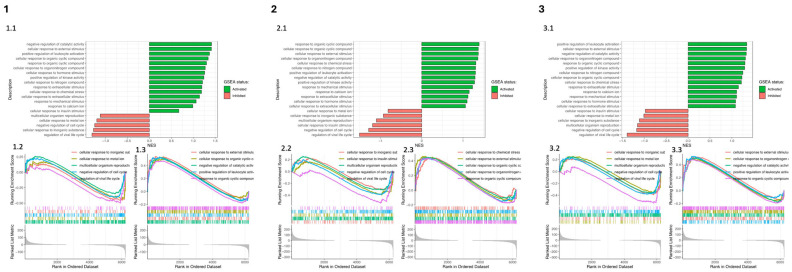
Gene Set Enrichment Analysis (GSEA) performed for cells at 48 h (**1**), 96 h (**2**), and 144 h (**3**) compared to the control (0 h). In (**1.1**,**2.1**,**3.1**), the normalized enrichment score (NES) is presented as a bar plot, with a positive NES indicating enrichment of a gene set at the top of a ranked list (denoted by green indicators), while gene sets with a negative NES are overrepresented at the bottom of the gene list (denoted by red indicators). In (**1.2**,**1.3**,**2.2**,**2.3**,**3.2**,**3.3**), detailed enrichment plots illustrate the profiles of the leading ES score and the positions of genes on the rank-ordered list for the top five inhibited/activated gene sets.

Overrepresented biological processes or pathways among the differentially expressed genes were identified (Figure 6). Based on the normalized expression level data, a list of significantly described terms from the Hallmark database software was provided. Only five (48 h) and six (96 h and 144 h) regulatory processes were inhibited in all groups analyzed. In particular, there were improvements in regulating vascular development and the cellular response to external stimuli.

**Figure 7:**
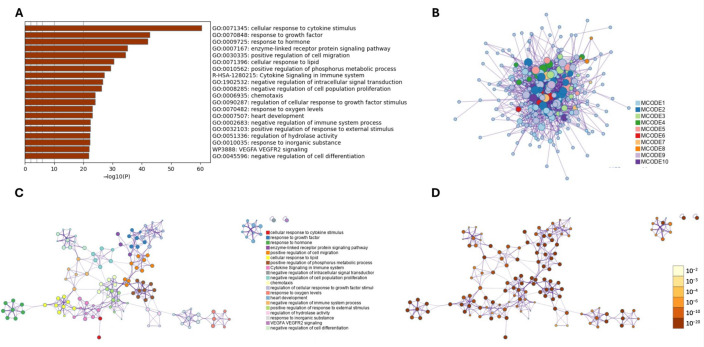
Functional enrichment analysis of differentially expressed genes (DEGs) using Metascape, clustered by biological process. (**A**) Bar chart showing the top 20 clustered enrichment ontology categories, based on the −log_10_(P) value, including GO and KEGG terms. (**B**) A Protein–Protein Interaction (PPI) network is organized into the top nine MCODE components. In this representation, each enriched Gene Ontology (GO) term was represented as a circular node, with the node’s size corresponding to the number of input genes associated with that term. Additionally, the color of each node indicates whether it belonged to a cluster or not. (**C**) Enrichment Ontology clusters are plotted, with each term represented as a circular node. The color of each node indicates the cluster identity to which the term belongs. (**D**) The enrichment network, where the nodes are colored on the basis of their *p*-values, which are presented in different shades, as indicated in the accompanying legend. Darker-colored nodes indicate higher statistical significance.

Additionally, an extensive functional enrichment analysis was performed utilizing the Metascape platform (Figure 7). Initially, overrepresented biological terms across all DEGs unveiled that biological processes are overrepresented in the input gene list.

In the top 20 statistically enriched GO terms, the top five were a cellular response to cytokine stimulus (GO:0071345, log_10_(P)= −60); response to growth factor (GO:0070848, log_10_(P)= −43); response to hormone (GO:0009725, log_10_(P)= −42); enzyme-linked receptor protein signaling pathway (GO:0007167, log_10_(P)= −35); and positive regulation of cell migration (GO:0030335, log_10_(P)= −34) (Figure 7A). To identify cohesive, functional clusters within the enrichment results, a sophisticated clustering algorithm was employed to cluster similar functional terms. These results are presented in a network layout to facilitate identifying related biological processes and pathways that may share common underlying mechanisms (Figure 7B–D).

**Figure 8:**
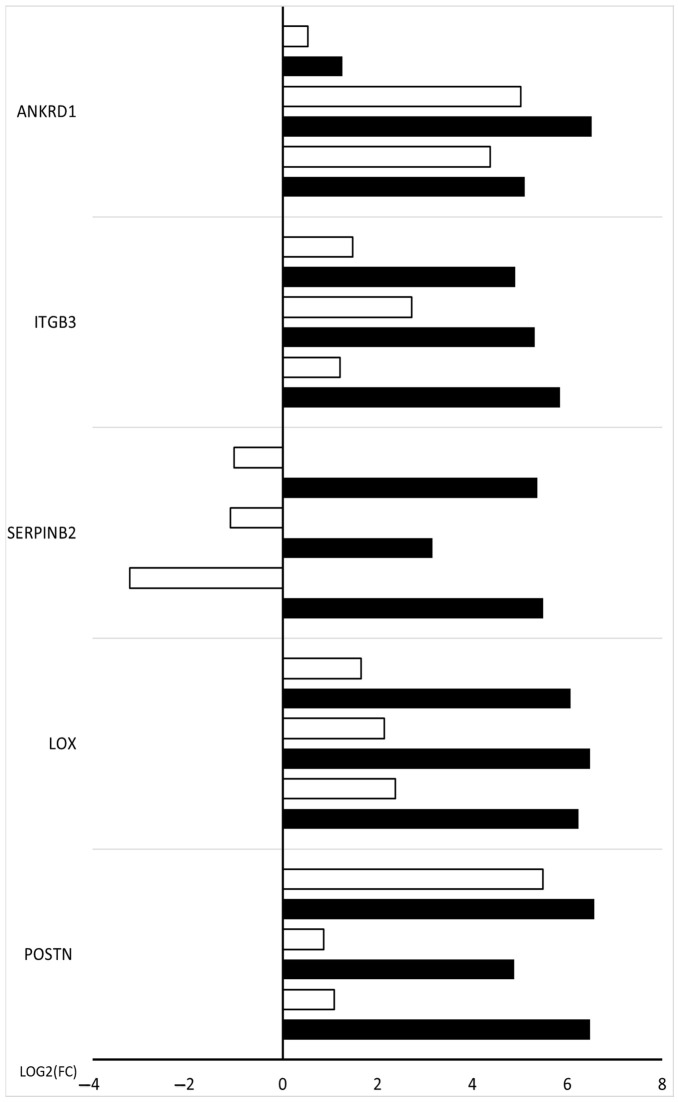
Quantitative validation of microarray data using RT-qPCR. The graph represents relative transcript levels in pGCs collected prior to cultivation (0 h; control group) versus three experimental groups (48 h, 96 h, and 144 h). The relative expression levels were calculated using the comparative 2^−ΔΔCT^ method. All analyzed groups showed statistically significant differences in gene expression compared with the control group (*p* < 0.05).

The microarray data were quantitatively validated using the RT-qPCR technique. A bar chart was created using the RT-qPCR assay data (Figure 8). The microarray analysis findings are displayed in the figure’s black bar, while the quantitative validation is displayed in the white bar. Significantly, four genes have had their direction of change measured. Only one gene displayed a change in expression direction that differed from what the expression microarray findings show. It was the *SERPINB2* gene. The difference in the direction of expression may result from different transcript variants in the microarray compared to those used in the design of primers for RT-qPCR. The RT-qPCR result is more reliable because all available transcript variants of the *SERPINB2* gene were used to design the primers. Moreover, RT-qPCR is a more quantitative method compared to the qualitative microarray method.

## 3. Discussion

This study investigated the changes in gene expression during in vitro culturing conditions at three time points (48 h, 96 h, and 144 h). The current findings reveal a variable and dynamic transcriptional program characterized by significant upregulation of genes associated with several key biological processes.

The analysis using GO technology highlighted five biological process terms consecutively enriched across all time points and one term visibly upregulated only at the 96 h time point of the experiment. The enrichment of the GO terms “cellular response to chemical stimulus” (GO:0070887) and “cellular response to organic substance” (GO:0010033) reflects not only intensified metabolic activity but also the activation of interconnected signaling pathways responsible for sensing and transducing extracellular stimuli into intracellular responses. Importantly, many of the identified genes appear to participate in common regulatory axes rather than acting independently. POSTN, LOX, and ITGB3 form functionally related ECM–integrin signaling modules involved in matrix organization, mechanotransduction, and cell survival. POSTN encodes periostin, a matricellular protein capable of binding integrins, including ITGB3-containing complexes, thereby activating focal adhesion kinase (FAK), PI3K/AKT, and MAPK signaling pathways associated with proliferation and survival of ovarian somatic cells. LOX-mediated collagen crosslinking further stabilizes the extracellular matrix and modifies its biomechanical properties, which may enhance integrin-dependent signaling and cytoskeletal tension sensing. Such coordinated ECM remodeling likely represents an adaptive response of GCs to attachment-dependent growth under in vitro conditions, and it may mimic early luteinization-associated structural reorganization observed in periovulatory follicles.

The significance of involvement of endogenous stimulation and chemical compound metabolism is also underlined via analysis of hierarchical clustering heatmaps. Rather than representing independent transcriptional events, the expression profile suggests the activation of an integrated adaptive regulatory network controlling pGCs survival, ECM remodeling, steroidogenesis, and stress adaptation during long-term in vitro culturing. The coordinated upregulation of *HSD3B1*, *POSTN*, *LOX*, *ITGB3*, and *SERPINB2* at 48 h and its maintenance at subsequent time points indicate the establishment of a stable transcriptional program enabling GCs to adapt to the in vitro environment. Another study, employing a mixture of antral follicle cells, suggests that the genes related to endogenous stimulation and catalytic processes, i.a., *HSD3B1* and *CYP11A1*, are upregulated during long-term in vitro culturing [59]. Upregulation of *HSD3B1* appears essential for progesterone synthesis [60]. *HSD3B1* is highly expressed in large ovarian follicles and regulated by hormonal signals such as LH, which induces its expression during luteinization, linking it to ovulatory changes and steroidogenic shifts [61,62,63]. The simultaneous upregulation of *HSD3B1* together with ECM-associated genes may indicate functional coupling between mechanotransduction and steroidogenesis. HSD3B1 is a key enzyme catalyzing progesterone biosynthesis, and it is strongly induced during luteinization. Steroidogenic activation in granulosa cells is not regulated exclusively by gonadotropins but also by ECM composition and integrin signaling, which modulate LH responsiveness and progesterone synthesis. Therefore, the coexistence of elevated *HSD3B1*, *POSTN*, *LOX*, and *ITGB3* expressions suggests that structural remodeling of the extracellular environment may actively support the acquisition of a steroidogenically active phenotype during prolonged culturing. Similar relationships between ECM remodeling and steroidogenic differentiation were reported in cultured ovarian follicles and luteinizing granulosa cells [62,63,64].

*POSTN*, *LOX* and *ITGB3* also promote GCs proliferation and follicular growth via adhesive potential pathways [65,66,67,68,69]. The significance of biological adhesion during in vitro culturing was also highlighted by Budna-Tukan et al. [70], who reveal the extended expression of genes related to adhesion, like *KRT18*, *GSN*, and *FN1*, in oviductal epithelial cells during long-term primary culturing.

On the other hand, *SERPINB2*, a serine protease inhibitor, may be involved in protecting GCs from cytolysis, also promoting its proliferation [71]. Also, the proliferation enhancement during in vitro culturing of GCs was proven by Kulus et al., highlighting upregulation of corresponding genes, like *HSD3B1*, *POSTN*, *LOX*, *ITGB3* and *SERPINB2* [72]. The next time point (96 h) showed significant increases in the expression of *ANKRD1*, *SLC1A1*, and *SFRP2*. The first of these, *ANKRD1*, particularly known in contexts of cancer [73,74], influences apoptosis by modulating endoplasmic reticulum (ER) stress, as suggested by other studies [75]. Similar results were obtained by Park et al., who reported that during in vitro maturation of cumulus–oocyte complex (COCs), the markers of ER stress increase [76]. Furthermore, Lin et al. describe ER stress modulation as a very important aspect in oocyte maturation and development [77]. *ANKRD1* interacts with key ER stress markers to regulate GCs survival, particularly in stress conditions such as those induced by hormonal fluctuations or an external stimulus [75]. Also crucial for stimuli responding, *SFRP2* has been shown to negatively regulate gonadotropin responsiveness, thereby attenuating FSH- and LH-mediated follicle survival and the number of maturing oocytes [78]. Gene expression upregulation was also reported by Chermuła et al., linking its functionality with angiogenesis [32]. Another gene indirectly linked with reproductive processes is *SLC1A1*, implicated in nutrient transport and supporting metabolic demand during follicular cell development [69]. Collectively, these genes illustrate the complex interplay of signaling pathways that balance proliferation, metabolism, intercellular regulations and steroidogenesis, ensuring proper follicle maturation and follicular dynamics.

The identified genes have a well-documented role in cellular stress responses associated with infection, pathological conditions, as well as adaptation to in vitro culturing. *POSTN*, *LOX*, *SERPINB2*, and *ITGB3* are strongly associated with extracellular matrix remodeling, inflammatory signaling, cell adhesion, and tissue repair processes that are frequently activated under conditions of cellular stress [79,80]. *POSTN* and *LOX* have been linked to hypoxia-induced remodeling and fibrosis-related responses, whereas *SERPINB2* is recognized as an important regulator of inflammation, apoptosis, and fibrinolytic balance during stress-associated cellular activation [81,82,83]. ITGB3 participates in integrin-mediated signaling and cell–matrix interactions that are essential for survival and adaptation of stressed cells [84]. In addition, ANKRD1 has been described as a stress-responsive transcriptional regulator induced by mechanical and oxidative stress, while SFRP2 is involved in Wnt-related signaling pathways associated with tissue remodeling and cellular adaptation [85,86]. The altered expression of *SLC1A1* and *HSD3B1* may further indicate metabolic reprogramming accompanying prolonged stress exposure and adaptation to changing environmental conditions [87,88]. Recent studies additionally emphasize the importance of host regulatory and transcriptional factors in stress adaptation. Wang et al. demonstrated that RBMX2 regulates infection-related stress transcriptional programs, like apoptosis, and epithelial remodeling [89]. Similarly, Xu et al. showed that ALKBH5-dependent m6A RNA modification modulates epithelial apoptosis and inflammatory responses during infection, thereby contributing to cellular survival and maintenance of epithelial integrity under infectious stress conditions [90]. Collectively, these observations suggest that the transcriptional alterations identified in the present study may represent conserved host adaptive mechanisms activated in response to environmental stressors.

Volcano plots confirmed the extensive transcriptional changes, revealing hundreds of up- and down-regulated genes at each time point. During the experiment, a specific pattern of increase in the number of genes with changed expression was noted, while, compared to 0 h, at 48 h there were a total of 1438 modified genes, and at 96 h as many as 2130 genes. At the last point of 144 h, the number of genes with modified expression dropped again, reaching 1.757 genes. This suggests that the most intense time of regulating endogenous stimulation and chemical compound metabolism during in vitro culturing is the time point of 96 h. The results reveal that gene expression changes in GCs, during in vitro culturing, show the most extensive fluctuations between 48 and 144 h of cultivation. Significant transcriptional adjustments at this compartment involve genes linked to proliferation, differentiation, ECM remodeling and survival-related changes. Similarly, Kocherova et al. revealed that primary pGCs apoptosis-related genes, such as *BCL2* and *BAX*, exhibit expression alterations on the 5th day of in vitro cultivation [91]. Additionally, as Kulus et al. reported, at the same time point, genes involved in structural remodeling, *KIF14* and *TACC3*, show peaks, supporting GCs function and survival in culture conditions mimicking native environments [92].

Pathway analysis using pathfindR revealed significant enrichment of proteoglycan-related signaling pathways at all time points of the experiment. Proteoglycans, especially cancer-related ones, represent diverse functions integral to physiological processes, including cell–cell and cell–ECM interactions, fundamentally influencing cell migration [93]. These functions are crucial for ovarian follicle development, specifically GCs organization around the oocyte within the COCs, supporting oocyte growth and maturation via intercellular communications and interactions [42]. Simultaneously, migration of theca cells contributes to follicular structural integrity, maturation, ovulation, and oocyte viability [94,95]. Furthermore, proteoglycans regulate growth factors and cytokine secretion and metabolism [96]. The observed proteoglycan pathway enrichment during our experiment may therefore reflect normal cellular processes during in vitro culturing rather than a neoplastic phenotype [97,98].

Further analysis of functional enrichment, using GSEA, shows that significant improvements in the regulation of vascular development and cellular response to external stimuli predominated. Only five of the analyzed biological processes were identified as inhibited during long-term in vitro culturing, including “cellular response to metal ion”, “cellular response to inorganic substance”, “response to insulin stimulus”, and “negative regulation of the cell cycle”. The reduced enrichment of processes associated with metal ions and inorganic substances may reflect the relatively isolated and chemically stable conditions of the in vitro environment. Under physiological conditions, pGCs are exposed to dynamic fluctuations of ions, metabolites, hormones, and paracrine factors originating from follicular fluid, theca cells, and systemic circulation. Many of these components, particularly calcium, zinc, iron, and magnesium ions, are crucial regulators of intracellular signaling, mitochondrial activity, oxidative balance, and steroidogenesis. Therefore, the diminished activation of these pathways may indicate reduced responsiveness of cultured pGCs to environmental ionic signaling due to the absence of the native ovarian microenvironment and limited diversity of extracellular stimuli in culture conditions [99].

Similarly, inhibition of insulin-responsive pathways may result from the restricted endocrine context of the culture system. In vivo, insulin cooperates with gonadotropins to regulate glucose uptake, proliferation, steroid hormone synthesis, and follicular growth. Reduced insulin-related signaling observed in the present study may therefore suggest gradual metabolic adaptation of granulosa cells to nutrient conditions maintained by the culture medium rather than by physiological endocrine regulation. Such adaptation may shift cellular metabolism toward maintaining survival and structural stability instead of intensive proliferative activity.

Finally, the Metascape analysis provided a comprehensive overview of enriched GO terms. The results reveal five of the mostly enriched GO terms, referring to specific cellular pathways, such as cellular response to cytokine stimulus (GO:0071345), response to growth factor (GO:0070848), response to hormone (GO:0009725), enzyme-linked receptor protein signaling pathway (GO:0007167) and positive regulation of cell migration (GO:0071310). The complex of mentioned pathways highlights the interplay of signaling pathways and cellular responses to external stimuli in the experimental environment [100,101,102].

Collectively, these findings indicate that the identified genes should not be interpreted as isolated biomarkers but rather as components of interconnected regulatory pathways integrating extracellular matrix remodeling, mechanotransduction, steroidogenesis, stress signaling, and metabolic adaptation. The temporal dynamics observed in the present study suggest that granulosa cells initially activate ECM- and adhesion-related adaptive mechanisms, followed by transcriptional programs associated with stress-response stabilization and metabolic homeostasis. Such sequential activation of interconnected pathways may constitute a fundamental mechanism enabling GCs to maintain viability and functional plasticity despite the absence of their native ovarian microenvironment.

The applied in vitro model, although allowing for precise control of experimental conditions, cannot fully reproduce the highly complex ovarian microenvironment in vivo. In physiological conditions, granulosa cells remain under continuous influence of endocrine signals, paracrine communication, ECM interactions, and signaling with surrounding follicular cells, all of which may substantially affect cellular behavior and gene regulation, but the transcriptomic fluctuations of pGCs ensure that long-term culturing provides valuable insights into GCs regulation.

Taken together, the complexity of the identified transcriptomic alterations indicates that additional studies integrating transcriptomics with proteomics, metabolomics, and functional cellular assays are necessary to better understand the molecular mechanisms regulating granulosa cell adaptation during long-term in vitro culturing. Future studies should focus on developing an understanding of these findings and clarifying the specific roles of the identified genes and pathways in the observed biological modulations, particularly in relation to the observed proteoglycanic enrichment.

## 4. Materials and Methods

### 4.1. Animal and Cell Isolation and Cultivation

A total of 40 crossbred Landrace gilts, with a median age of 170 days and an average body weight of 98 kg, were utilized in this study. All animals were maintained under standardized environmental and management conditions. The gilts attained sexual maturity within the expected physiological range, as swine typically reach puberty between 4 and 6 months of age.

Ovaries (*n* = 80) and entire reproductive tracts were collected at slaughter and transported to the laboratory at 38 °C in a 0.9% NaCl solution within 30 min. The ovaries exhibited an ovoid shape with a median length of 4.2 cm. Their external surface was irregular and nodular, resembling the texture of a mulberry fruit. The appearance of the ovarian surface was influenced by the presence of ovarian follicles distributed within the parenchymatous zone. This zone was enveloped by the tunica albuginea and an outer layer of superficial epithelium. The ovarian medulla, composed of connective tissue, contained an extensive vascular network.

**Figure 9:**
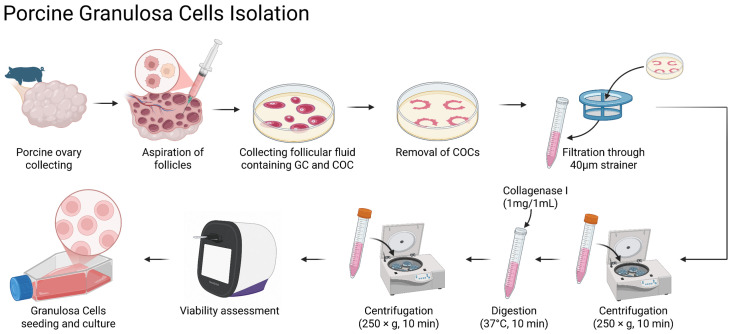
Workflow of granulosa cell isolation and in vitro culture.

Granulosa cells were isolated due to the following protocol, as showed at Figure 9. The ovaries from each animal were placed in phosphate-buffered saline (PBS) (Sigma-Aldrich, Saint Louis, MO, USA). Subsequently, single pre-ovulatory large follicles with an estimated diameter greater than 5 mm (*n* = 300) were aspirated into a sterile Petri dish by puncturing with a 5 mL syringe and a 20 G needle. The procedure allowed for the recovery of cumulus–oocyte complexes COCs and follicular fluid (FF). The follicular fluid was then used for the isolation of GCs, while the COCs were discarded. After discarding COCs, the extracted FF was filtered through sterile cell strainers with a pore diameter of 40 µm (Biologix Group, Jinan, China) to eliminate tissue debris and larger cell aggregates, including aggregates from the blood constituents. The filtrate was then centrifuged (room temperature; 10 min; 200 rpm) to obtain a GCs cellular pellet. The pellet then was resuspended in a collagenase type I solution (Gibco, Thermo-Fischer Scientific, Waltham, MA, USA) (1 mg/1 mL DMEM) and incubated in a water bath (10 min in a 37 °C) and centrifuged (under the above-mentioned conditions).

GCs were cultured in a culture flask (25 cm^2^; Genoplast Biochemicals, Rokocin, Poland), with the assumption that the number of cells was seeded at 3 × 10^6^ cells per flask. The number of cells and their viability were assessed using an ADAM automatic cell counter (NanoEnTek, Waltham, MA, USA), and for further analysis only samples with a cell viability above 85% were used. The basic medium was Dulbecco’s Modified Eagle’s Medium (DMEM, Sigma-Aldrich, Saint Louis, MO, USA) with fetal bovine serum (FBS) (2%) (Sigma-Aldrich, Saint Louis, MO, USA), L-glutamine (200 mM) (Invitrogen, Carlsbad, CA, USA), gentamicin (10 mg/mL) (Invitrogen, Carlsbad, CA, USA), penicillin (10,000 units/mL) (Invitrogen, Carlsbad, CA, USA), and streptomycin (10,000 μg/mL) (Invitrogen, Carlsbad, CA, USA). The cells were cultured under the conditions of 38 °C and 5% CO_2_. The cells were passaged after reaching more than 80% confluence, using trypsin-EDTA (0.05%) (Invitrogen, Carlsbad, CA, USA). Cells were cultivated until culture termination, and the material was collected at 0 h, 48 h, 96 h, and 144 h. The culture medium was changed every 72 h. For each experimental group biological replicates, two replicates for microarray expression, and triplicate samples for RT-qPCR were maintained [72].

### 4.2. Microarray Analysis

#### 4.2.1. Microarray Expression Study

To explore the transcriptome analysis the total RNA from porcine granulosa cells was assembled from two independent replicates from each experimental variant: control—0 h, 48 h, 96 h, and 144 h of the experiment, according to other studies that analyzed GCs gene expression at various time points [33,92]. Selected time points were designed to possibly preserve native cell morphology because, as shown in the above articles, long-term culturing changes the morphological profile of the cell, depriving it of its native tissue context. Each replicate includes RNA from three independent experiments. The microarray study was performed according to the previously described protocol [103]. First, the total RNA (100 ng) from each sample was submitted to a two-step cDNA synthesis reaction, biotin labeling, and fragmentation according to the manufacturer’s instructions (GeneChip^®^ WT Plus Reagent Kit, Affymetrix, Santa Clara, CA, USA). Then the biotin-labeled fragments of cDNA were hybridized to the PorGene 1.1 ST Array Strip (Affymetrix^®^, Santa Clara, CA, USA) (45 °C/20 h). Next, the microarrays were stained by the Affymetrix GeneAtlas Fluidics Station of the GeneAtlas System. The microarrays were scanned by the Imaging Station of the GeneAtlas System (Affymetrix, Santa Clara, CA, USA). The Affymetrix GeneAtlas Operating System was performed for the analysis of the obtained results. The quality of gene expression data was confirmed using the software’s quality control criteria.

#### 4.2.2. Microarray Data Analysis

The BioConductor (version 3.19) software with the relevant Bioconductor libraries, by the statistical R programming language (v4.1.2; R Core Team 2021), was used to perform all analyses. For the normalization, background correction, and calculation of the expression values of the analyzed genes, the robust multiarray average (RMA) normalization algorithm implemented in the “Affy: library” was applied [104]. To show the total number of up- and down-regulated genes, a principal component analysis (PCA) of the filtered data set was performed and visualized using the “factoextra” library [105]. Next, the DAVID (Database for Annotation, Visualization, and Integrated Discovery) bioinformatics tool was used for functional annotation and clusterization of differentially expressed genes (DEGs) [106]. For DEGs the established cut-off criteria were based on the differences in the absolute value from the expression fold change |FC| > 2 (i.e., an at least 2-fold upregulation or down-regulation relative to the control at 0 h). Moreover, the expressed genes were assigned to relevant GO terms, with the subsequent selection of significantly enriched GO terms using the GO BP DIRECT database. *p*-values for GO term enrichment were corrected using the Benjamini–Hochberg false discovery rate (FDR) procedure; only GO terms with corrected *p* < 0.05 and a minimum of >2 genes per group were considered significantly enriched [107]. Next, DEGs from each comparison were visualized as a heatmap with hierarchic clustering of differentially expressed genes using the “ComplexHeatmap” library [108]. Genes belonging to the first nine most significantly enriched ontological groups (lowest adjusted *p*-value) are shown on the figures with the expression values of analyzed genes.

GSEA was performed by using the “clusterProfiler” Bioconductor library [109] to identify the level of depletion or enrichment in GO terms by calculation of normalized enrichment score (NES) with relevant *p*-values. Normalized fold change values from all of the genes were log2 transformed, sorted, and used as an argument for the “gseGO” function. Gene set enrichment was performed with reference to the “biological process” GO category, assuming that the minimum size of each geneSet for analyzing = 50 and a *p*-value cut-off = 0.05. Then hierarchical clustering of enriched terms based on pairwise similarity calculation with Jaccard’s similarity index was performed. The result of the analysis qualified individual GO terms to clusters based on their functional similarity. The obtained clusters are presented as tree plots. The ten ontology groups with the highest enrichment score (the highest NES value) and the 10 groups with the most depleted enrichment score (the lowest NES value) are visualized as a bar chart. Enrichment plots for five of the most enriched and depleted GO terms are also presented. Next, the genes were mapped onto the “ Inflammatory response pathway”, “Overview of proinflammatory and profibrotic mediators pathways”, and “Clonal expansion of T cells pathway in human granulosa cells” from the WikiPathways database [110] using the “rWikiPathways” [110] library and Cytoscape (version 3.10.2) software [111].

Metascape was used as the method to identify functional protein patterns [112]. Metascape serves as a comprehensive resource for analyzing and interpreting gene and protein function, pathway analysis, and protein–protein interaction (PPI) network analysis. For the PPI network analysis, the minimum required interaction score was set to medium confidence (0.4). When the PPI network consisted of more than three nodes, the Detection (MCODE) algorithm was applied to uncover clusters that were directly associated with genes within the network [113]. Additionally, MCODE assigned a unique color to each cluster based on the *p*-value, providing further insights into the generated network.

### 4.3. RNA Isolation

Total RNA was extracted at 48, 96, and 144 h cell culture initiation. The cells underwent treatment analogous to passaging, after which the cell pellet was resuspended in 1 mL of the TRI Reagent Solution (TRI Reagent^®^, SIGMA-ALDRICH, St. Louis, MO, USA, Merck KGaA, Darmstadt, Germany). The samples were subsequently stored at −80 °C until RNA isolation.

Total RNA was isolated following the Chomczyński and Sacchi protocol [114]. Initially, 0.2 mL of chloroform (SIGMA-ALDRICH, St. Louis, MO, USA, Merck KGaA, Darmstadt, Germany) was added to the samples, which were then mixed by inversion and shaken for 15 s, followed by a 15 min incubation at room temperature. The biphasic emulsion was subsequently separated via centrifugation at 12,000× *g* for 15 min at 4 °C. The upper aqueous phase containing RNA was carefully transferred to new Eppendorf tubes. Following this, 0.5 mL of isopropanol (SIGMA-ALDRICH, St. Louis, MO, USA, Merck KGaA, Darmstadt, Germany) was added, and the samples were mixed by inversion, shaken for 15 s, and incubated for 10 min at room temperature. The samples were then centrifuged at 12,000× *g* for 10 min at 4 °C. The resulting precipitate was washed with 1 mL of a 75% ethanol solution (SIGMA-ALDRICH, St. Louis, MO, USA, Merck KGaA, Darmstadt, Germany), vortexed for 20 s, and centrifuged at 7500× *g* for 15 min at 4 °C. After discarding the supernatant, the samples were air-dried and subsequently dissolved in 10–20 µL of DEPC-treated water, depending on the pellet size.

Spectrophotometric analysis at λ = 260 nm was conducted using a NanoDrop spectrophotometer (Thermo Fisher Scientific, Waltham, MA, USA) to determine the concentration of the RNA samples.

### 4.4. Reverse Transcription–Quantitative Polymerase Chain Reaction (RT-qPCR)

A quantity of 250 ng of isolated RNA, diluted in PCR-grade water to a final volume of 8 µL, was reverse-transcribed using the RT2 First Strand Kit (Qiagen^®^, Hilden, Germany) according to the manufacturer’s protocol. Except during incubation periods, the samples were kept on ice. To eliminate genomic DNA, 2 µL of GE (5× *g* DNA Elimination Buffer) was added to 1 µg of isolated RNA, followed by incubation at 42 °C for 5 min. Subsequently, the reaction mixture, consisting of 4 µL of BC3 (5× RT Buffer 3), 1 µL of P2 (Primer and External Control Mix), 2 µL of RE3 (RT Enzyme Mix 3), and PCR-grade water to reach a final volume of 10 µL, was prepared. The samples then underwent two incubation steps: 15 min at 42 °C and 5 min at 95 °C. After incubation, the samples were cooled on ice, and 91 µL of H_2_O was added to each reaction.

Validation of the microarray data was conducted using a LightCycler^®^ 96 Instrument (Roche Diagnostics GmbH, Mannheim, Germany), with cDNA synthesized during reverse transcription serving as the template. Primers were designed using the Primer3Plus software (version 0.4.0; Whitehead Institute for Biomedical Research, Massachusetts Institute of Technology, Cambridge, MA, USA) based on sequences of selected transcript variants of the genes available in the Ensembl database (Table 1 and Table 2) and NCBI RefSeq database (Table 2). The reaction mix components included the QUANTUM EvaGreen^®^ PCR Kit (5×) (Syngen Biotech, Wroclaw, Poland) as the master mix, 10 µM of oligodeoxynucleotides (SIGMA-ALDRICH, St Louis, MO, USA, Merck KGaA, Darmstadt, Germany), and PCR-grade water. A total of 9 µL of the reaction mix and 1 µL of the template were added to each well of a 96-well plate. The plate was then sealed with a sealing foil, centrifuged at 1500 rpm (400× *g*) for 1 min, and placed in a thermocycler. The PCR amplification conditions consisted of an initial denaturation at 95 °C for 10 min, followed by 40 cycles of 95 °C for 15 s and 60 °C for 60 s. A melting curve analysis (65–95 °C) was performed at the end of each run to verify single-amplicon specificity. To ensure compliance with the MIQE guidelines, all experimental groups were analyzed using 3 independent biological replicates (separate cell cultures), and each sample was run in 3 technical replicates. Target gene expression was normalized against three stably expressed reference genes: ACTB, GAPDH, and HPRT. The relative expression levels were calculated using the comparative 2^−ΔΔCT^ method.

## 5. Conclusions

Culturing of GCs reveals dynamic transcriptomic changes characterized by extensive upregulation of genes involved in cellular response to endogenous and external stimuli, chemical compound metabolism, and processes related to vascular development and GCs. The *HSD3B1*, *LOX*, and *POSTN* transcriptomic changes suggest coordinated and interconnected regulatory mechanisms underlying intercellular communication. The influenced processes revealed GCs’ sensitivity to changes in the culture environment. Subsequently, processes related to facing ER stress and apoptosis, as well as those supporting proliferation, appear to play an important role in regulating GCs. The main changes through the time points are related to intercellular communication and transitions aimed at adapting to environmental conditions.

Further investigation into the interplay between the pathways will provide a more comprehensive understanding of the role of GCs in the reproductive system, especially folliculogenesis, oocyte maturation and ovulation.

## Figures and Tables

**Table 1:** Oligonucleotide sequences of primers used for RT-qPCR analysis.

Gene	Primer Sequence (5′−3′)		Product Size (bp)
*LOX*	F	GTACAACCTGAGATGCGCTG	208
	R	GCTGAATTCGTCCATGCTGT	
*ANKRD1*	F	CTGCTTGAGGTGGGGAAGTA	178
	R	GTGTCTCACTGTCTGGGGAA	
*ITGB3*	F	GGCTTCAAAGACAGCCTCAC	175
	R	AGTCCTTTTCCGAGCACTCA	
*POSTN*	F	ATTGACCGTGTCCTCACACA	212
	R	GCCACTTTGTCTCCCATGAT	
*HSD3B1*	F	TCCACACCAGCAGCATAGAG	245
	R	CATGTGGGCAAAGATGAATG	
*SERPINB2*	F	GGAAGAATACATTCGACTCTCCA	170
	R	TGGTCTCCGCATCTACAGAA	
*ACTB*	F	CCCTTGCCGCTCCGCCTTC	156
	R	GCAGCAATATCGGTCATCCAT	
*GAPDH*	F	CCAGAACATCATCCCTGCCT	185
	R	CCTGCTTCACCACCTTCTTG	
*HPRT*	F	CCATCACATCGTAGCCCTC	166
	R	ACTTTTATATCGCCCGTTGAC	

**Table 2:** Characteristics of primers and NCBI RefSeq mRNA accession numbers for RT-qPCR validation.

Gene Symbol	Gene Name	NCBI Gene ID	Role in Study
*LOX*	Lysyl Oxidase	NM_001244335	Target Gene
*ANKRD1*	Ankyrin Repeat Domain 1	NM_214227	Target Gene
*ITGB3*	Integrin Subunit Beta 3	NM_214154	Target Gene
*POSTN*	Periostin	NM_001244321	Target Gene
*HSD3B1*	Hydroxy-delta-5-steroid dehydrogenase, 3 beta- and steroid delta-isomerase 1	NM_001006652	Target Gene
*SERPINB2*	Serpin Family B Member 2	NM_001244111	Target Gene
*ACTB*	Actin Beta	NM_001101841	Reference Gene
*GAPDH*	Glyceraldehyde-3-Phosphate Dehydrogenase	NM_001206359	Reference Gene
*HPRT1*	Hypoxanthine Phosphoribosyltransferase 1	NM_001032446	Reference Gene

## Data Availability

The original contributions presented in this study are included in the article. Further inquiries can be directed to the corresponding author.
